# Galectin-9 Mediates the Therapeutic Effect of Mesenchymal Stem Cells on Experimental Endotoxemia

**DOI:** 10.3389/fcell.2022.700702

**Published:** 2022-02-17

**Authors:** Yiming Zhao, Dingding Yu, Hongda Wang, Wang Jin, Xiang Li, Yonghao Hu, Yafei Qin, Dejun Kong, Guangming Li, Acheampong Ellen, Hao Wang

**Affiliations:** ^1^ Department of General Surgery, Tianjin Medical University General Hospital, Tianjin, China; ^2^ Department of Hepatobiliary Surgery, Union Hospital, Tongji Medical College, Huazhong University of Science and Technology, Wuhan, China; ^3^ Tianjin General Surgery Institute, Tianjin, China; ^4^ Department of Pathology, Massachusetts General Hospital, Boston, MA, United States

**Keywords:** galectin-9, mesenchymal stem cell, lipopolysaccharide, endotoxemia, immunoregulation

## Abstract

Endotoxemia remains a major cause of mortality in the intensive care unit, but the therapeutic strategy is still lacking. Mesenchymal stem cell (MSC) was reported with a tissue-oriented differentiation ability and an excellent immunoregulatory capacity. However, the immunity signaling pathways that govern MSC modulation effect are not completely understood. In our current study, MSCs (2.5 × 10^5^ /ml) were obtained and stimulated with IFN-γ (20 ng/ml) for 72 h. Gal-9 expression on MSCs was measured by ELISA, RT-PCR, flow cytometry, and immunofluorescence, respectively. Experimental endotoxemia was induced by LPS injection (10 mg/kg, i. p.) followed by the treatment with Gal-9 high-expressing MSCs, unmodified MSCs, and Gal-9 blocking MSCs. Therapeutic effects of MSCs were assessed by monitoring murine sepsis score, survival rate, splenocyte proportion rate, inflammatory mediator levels, and pathological manifestations. The results showed that Gal-9 expressed in MSCs, and this expression was increased in a dose-dependent manner after pre-stimulating with IFN-γ. Adoptive transfer of Gal-9 high-expressing MSCs into modeling mice significantly alleviated endotoxemia symptoms and multi-organ pathological damages. Splenocyte analysis indicated that Gal-9 high-expressing MSCs could promote macrophage polarization to M2-subtype and boost Treg generation. Moreover, there were also attenuated pro-inflammatory mediator expressions (TNF-α, IL-1β, IFN-γ, and iNOS), and increased anti-inflammatory mediator expressions (T-SOD and IL-35) in the sera and damaged organ homogenates. Additionally, we found a higher expression of Gal-9 in liver, lung, and kidney homogenate. Taken together, this study reveals that the optimized immunoregulatory effect of MSCs is strongly correlated with Gal-9 high expression, which provides a novel idea for the investigation of MSC immunomodulatory mechanisms and offers a potential strategy for the treatment of endotoxemia in clinical settings.

## Introduction

Sepsis is regarded as a major public health concern and accounts for 19.7% of all global deaths annually (Singer et al., 2016; Rudd et al., 2020). Endotoxemia is an important pathophysiological process of sepsis and mediated by uncontrolled immunocytes activation toward lipopolysaccharide (LPS) (Rittirsch et al., 2008; Dickson and Lehmann, 2019). Endotoxemia could lead to the severe septic shock with production of a large number of biologically active substances, including cytokines, bioactive amines, and reactive oxygen species, and thus lethal to the hosts (Andrades et al., 2011).

Until now, a majority of clinical therapies implemented are mainly focused on supporting treatments, including incentive removal, antibiotics application, circulatory resuscitation, mechanical ventilation, and renal replacement (Perner et al., 2016). Although those strategies are effective when implemented timely and appropriately, they are often considered inadequate due to the lack of immunomodulatory intervention. Several clinical trials have tried to adopt monoclonal antibody to neutralize cytokines or block inflammatory factor receptors (Calandra et al., 2000). However, these attempts were proven with limited effects due to only targeting a few receptors (Ianaro et al., 2009; Opal et al., 2013). Therefore, seeking a novel therapeutic method to supplement the existing immunomodulatory strategy is in urgent need.

Mesenchymal stem cell (MSC), a newly discovered pluripotent stem cell, was reported with multiple differentiation ability, self-renewal capacity, and immunoregulation specialties (Fan et al., 2020). MSCs express low levels of histocompatibility complex (MHC) I and MHC II molecules on their surface, indicating that MSCs could be well-accepted by recipients (Di Nicola et al., 2002; Le Blanc et al., 2003). It has also been reported that MSCs could interrupt continuous damages in organs and promote tissue repair (Fu et al., 2019). Given these encouraging characters, MSC-based cell therapy has been explored in various pre-clinical and clinical studies, such as multiple sclerosis, rheumatoid arthritis, Crohn’s disease, ischemic kidney injury, and systemic lupus erythematosus (Le Blanc et al., 2004; Ménard and Tarte, 2013; Galipeau and Sensébé, 2018).

Of interest, although the promising function of MSCs has been widely recognized, the specific molecular mechanism underlined is not fully understood. Growing evidences have suggested that MSCs could modulate the immune response through direct cell-to-cell contact or by regulators secretion, such as NO, IL-10, TGF-β, VEGF, PGE2, and indoleamine-2,3-dioxygenase (IDO) (Kang et al., 2008; Spees et al., 2016; Fan et al., 2020). Meanwhile, a series of studies has also revealed that IFN-γ was required for MSC activation. However, the vital phenotype changes in IFN-γ stimulated MSCs remain to be elucidated (Krampera et al., 2006; Polchert et al., 2008).

Inspired by previous reports and preliminary data, we found that MSCs could express Galectin-9 (Gal-9), and proved that Gal-9 expression increased in a dose-dependent manner after being stimulated by IFN-γ. Gal-9, an important member of the galectin family, with two carbohydrate recognition domains (CRDs) joined by a linker peptide, and is a natural ligand for T-cell immunoglobulin mucin-3 (Tim-3) (Cao et al., 2007). While Tim-3, one of the wildly studied immune checkpoints, was documented to be expressed in Th1/17 cells, CD8^+^ cytotoxic T cells, regulatory T cells (Treg), macrophage, and natural killer (NK) cells (Anderson et al., 2016). When Gal-9 binds to Tim-3^+^ cells, it can negatively regulate Th1 and Th17 immunity (Zhu et al., 2005), promote macrophage polarization to the M2 subtype (Lv et al., 2017; Li et al., 2018), and boost Foxp3^+^ Treg generation (Seki et al., 2008).

Given together, the present study was designed to explore whether MSCs express Gal-9 and whether Gal-9 is involved in the therapeutic effects of MSCs in alleviating endotoxemia.

## Materials and Methods

### Animals

Male C57BL/6 mice weighing 18–20 g and aged 6–8 weeks were purchased from the China Food and Drug Inspection Institute (Beijing, China). All the mice were fed with a standard diet and acclimated in the animal care facility for 7 days before experiments. Experiments involving animals all complied with the standard protocols (IRB2020-DW-02) approved by the Animal Care and Use Committee of Tianjin Medical University (Tianjin, China), according to the Chinese Council on Animal Care guidelines.

### MSC Preparation

Mesenchymal stem cell used in this experiment came from inguinal fat and is referred to as “adipose derived mesenchymal stem cell (AD-MSC)”. The obtained procedure was in accordance with the methods described previously (Araña et al., 2013). Briefly, a 6-week-old mouse was sacrificed first and sterilized in 75% alcohol for 5 min. Then, bilateral inguinal fat was extracted and shred on the ultra-clean workbench. Fragment fat tissues were soaked in DMEM medium (Hyclone Laboratories Inc., United States) containing 1 mg/ml collagenase type I (Solarbio, Beijing, China) and digested in a shaker (37°C, 5% CO_2_) at 300 rpm for 30 min. After digestion, pellet cells were harvested and suspended in the DMEM-F12 culture medium with 10% FBS and 1% penicillin/streptomycin. The culture medium was changed every 2 days to remove the non-adherent cells. When the cells expand to 80% of the plate, the adherent cells were passaged down at a ratio of 1:3, and finally the MSCs were harvested for a purity test and subsequent experiments.

### Detection of Gal-9 Expression in AD-MSCs

The obtained and purified AD-MSCs were passaged down. The expression of secreted Gal-9 was detected in second to fifth generation MSC (2.5×10^5^ /ml) culture supernatant. Furthermore, the third generation of MSCs (2.5×105 /ml) was cultured in 6-well plates (DMEM-F12 culture medium with 10% FBS and 1% penicillin/streptomycin) and added with different concentrations of IFN-γ (0–20 ng/ml, 72 h, PeproTech, Rocky Hill, United States) for stimulation. After which, the total protein of MSCs was extracted by RIPA lysis buffer (Solarbio, Beijing, China). Then, the culture supernatant and MSC protein were collected to determine Gal-9 expression.

In addition, Gal-9 expression in the third generation of AD-MSCs (2.5 × 10^5^ /ml, 20 ng/ml IFN-γ primed) was analyzed by flow cytometry and RT-PCR.

The third generation of AD-MSCs (2.5 × 10^5^ /ml) was further stimulated with 20 ng/ml IFN-γ for 72 h and cultured for an additional 24 h after washing away the stimulators. ELISA and RT-PCR were performed to test Gal-9 protein and mRNA expression, respectively. In addition, to reduce the systemic error, we have also performed two technical replicates for each sample.

### Immunofluorescence Staining

Third-generation AD-MSCs (2.5×10^5^ /ml) were obtained and seeded in a 6-well plate with or without IFN-γ stimulation (20 ng/ml). After culturing for 72 h, these crawled cells were washed with PBS for three times and fixed in a 4% (w/v) paraformaldehyde solution for 15 min, followed by permeabilizing in 0.1% Triton X-100 solution for 5 min. After blocking with BSA, anti-mouse Gal-9 primary antibody (Abcam, 1:1000 dilution) was adopted to incubate the slides at 4°C overnight. On the other day, Alexa Fluor® 488-conjugated secondary antibody (Jackson ImmunoResearch Inc., United States) was further used to incubate the slides in a dark cassette for 60 min, followed by mounting with antifade mountant containing DAPI (SouthernBiotech, United States). Finally, these slides were obtained and photographed under fluorescence microscope (Olympus, Japan).

### Experimental Groups and Endotoxemia Induction

Mice were randomly divided into five experimental groups (*n* = 6) and received lipopolysaccharide (LPS, 10 mg/kg, i.p.) or adipose derived-MSC (500 ul, i.v.) injection at 0 and 1 h time points: (1): Normal control group: Receiving 500 ul PBS (i.v.) (2). Untreated group: Endotoxemia model was induced by LPS injection (10 mg/kg, i. p.) (Solarbio, Beijing, China), followed by PBS (500 ul) injection without MSCs (3). Unmodified MSC group: After model establishment, mice were lightly anesthetized by inhalation with isoflurane. 10^6^ MSCs diluted in 500 ul PBS were injected through the penile dorsal vein (4). Gal-9 high-expressing group: MSCs were co-cultured with IFN-γ solution (20 ng/ml) (PeproTech, Rocky Hill, United States) for 72 h in advance. When it is time to inject, MSCs were accurately counted at 10^6^ and dissolved in 500 ul PBS for intravenous injection (5). Gal-9 blocking group: 10^6^ MSCs was diluted in 500 ul PBS containing 10.8 mg/ml α-lactose (α-lactose: the specific antagonist of Gal-9, Sigma-Aldrich), and then used for injection.

All mice were sacrificed 24 h later. Spleen were collected for flow cytometry analysis (detailed procedure is shown below). Serum, and half parts of liver, lung, and kidney were frozen in −80°C for further analysis. Half parts of liver, lung, and kidney were immersed in formalin solution for pathological analysis.

### Survival Observation and Clinical Symptom Evaluation

The health state of the mice (*n* = 8) was monitored by two investigators every 6 h. Both investigators were blinded with the treatment information and were requested to assess the condition of the mice in accordance with the criteria reported previously (Shrum et al., 2014). Briefly, murine sepsis score (MSS) evaluation system mainly includes the following aspects: appearance, spontaneous activity, eyes condition, level of consciousness, response to stimuli, respiration rate, and respiration quality. Each of these variables was given a score between 0 and 4, while the total score summed up was 0–28.

### T- SOD Activity and MDA Content Measurement

Serum total superoxide dismutase (T-SOD) activity and malondialdehyde (MDA) content were assayed by the hydroxylamine and modified 2-thiobarbituric acid (TBA) spectrophotometry method. The procedures performed were all in accordance with the manufacturer’s instructions (Nanjing Jiancheng Bioengineering Institute, Nanjing, China). Each assay included three parallel samples.

### Enzyme-Linked Immunosorbent Assay

The culture supernatant of second to fifth generation MSCs, and MSCs with or without IFN-γ stimulation were collected to test Gal-9 expression. Furthermore, the liver, lung, and kidney tissue homogenate were also obtained to detect Gal-9 expression level (BaoLai biological technology, Jiangsu, China). In addition, the serum and liver homogenate were gathered, respectively, to detect the expression level of TNF-α and IL-1β (DAKEWE, Shenzhen, China). According to the manufacturer’s instructions, the detection antibody, HRP conjugate, chromogenic substrate, and stop solution were added in order. Finally, the absorbance of each well was detected at 450 nm, and the concentration of each sample was obtained by comparing with the standard curve. All tests were performed in duplicate to eliminate the error.

### Flow Cytometry Analysis

For flow cytometry staining, cell suspensions of MSCs with or without IFN-γ stimulation were prepared. Among which, half of the MSC suspensions were incubated with fixation and permeabilization buffer (Thermo Fisher Scientific, United States). Then all the specimens were incubated with Gal-9 primary antibody (Thermo Fisher Scientific, United States) for 30 min, followed by staining Alexa Fluor® 488-conjugated secondary antibody for 30 min in a dark place. Last, all samples were washed three times and analyzed on the FACS cytometer (BD Biosciences, United States).

In addition, the spleens from each group were obtained separately, and then ground with a 100-mesh filter to make a single-cell suspension. After disposing with blood cell lysing buffer (Solarbio, Beijing, China), these suspensions were washed and dispensed in tubes (100 μl) for further staining. Fluorescent monoclonal antibodies FITC-CD68^+^(137006), PE-CD206^+^(141706), FITC-CD4^+^(11-0042-82), PE-CD25^+^(12-0862-82), and PE-Cy5-Foxp3^+^(15-5773-82) (Biolegend; ebioscience Inc.) were used for staining M2-type macrophage (CD68^+^CD206^+^), total macrophage (CD68^+^), and Tregs (CD4^+^CD25^+^Foxp3^+^). The average number of collected splenocytes was 150,000 and we present the data here in proportion. As for the gating strategy, all the collected splenocytes were gated. CD68^+^ cells were selected for total macrophage and CD68^+^CD206^+^ cells were determined for M2 subtype. As for Treg cells, splenocytes and FITC-CD4^+^ cells were first gated, and then PE-CD25 and PE-Cy5-Foxp3 double positive cells were detected for Tregs. All the data acquired were analyzed by flowjo V10 software.

### Pathological Analysis

Liver, lung, and kidney tissues fixed in 10% formalin were obtained, respectively. After undergoing paraffin embedding, sectioning, dehydration, and HE staining, all the specimens were sealed with neutral gum, and observed under microscope. All sections were scored and evaluated by two pathologists after double-blind reviewing. The scoring criteria for evaluating liver damage are as follows: necrosis, sinus congestion and edema, lipid vacuoles and infiltration of hyperemia, and inflammatory cells. Each item has a score of 0–4, and the total score is 0–16 (Inata et al., 2018). While the criteria for assessing kidney injury are as follows: tubular dilatation/flattening, tubular casts, and tubular degeneration/vacuolization (cortex and medulla, 0–3, total: 0–18) (Wang et al., 2009). In addition, criteria for evaluating lung damage are as follows: edema, alveolar and interstitial inflammation, alveolar and interstitial hemorrhage, and necrosis (0–4, total: 0–16) (Smith et al., 1997).

### Real-Time Polymerase Chain Reaction

The above-obtained MSCs, in addition to liver, lung, and kidney tissue homogenate, were collected to extract total RNA by the commercial RNA extraction kits (DP430, DP431, Tiangen Biotech Co. Ltd.). To determine purity and concentration, the extracted RNA was evaluated with a UV spectrophotometer at the spectrum of 260 and 280 nm. Then, cDNA was acquired from the obtained RNA by reverse transcribed method with a FastKing one-step kit (KR106, Tiangen Biotech Co. Ltd). RT-PCR reaction was carried out according to the manufacturer’s recommended instruction by SuperReal Color Premix kit (FP216, Tiangen Biotech Co. Ltd.). The primer sequences of all reactions were designed as follows:

Gene Primers (5′-3′)
  Gal-9
    Forward: ATG​CCC​TTT​GAG​CTT​TGC​TTC
    Reverse: AAC​TGG​ACT​GGC​TGA​GAG​AAC
  TNF-α
    Forward: CCC​TCA​CAC​TCA​GAT​CAT​CTT​CT
    Reverse: GCT​ACG​ACG​TGG​GCT​ACA​G
  IL-1β
    Forward: TTC​AGG​CAG​GCA​GTA​TCA​CTC
    Reverse: GAA​GGT​CCA​CGG​GAA​AGA​CAC
  IFN-γ
    Forward: ATG​AAC​GCT​ACA​CAC​TGC​ATC
    Reverse: CCA​TCC​TTT​TGC​CAG​TTC​CTC
  IL-35 (EBI3)
    Forward: CTT​ACA​GGC​TCG​GTG​TGG​C
    Reverse: GTG​ACA​TTT​AGC​ATG​TAG​GGC​A
  SOD
    Forward: CAG​ACC​TGC​CTT​ACG​ACT​ATG​G
    Reverse: CTC​GGT​GGC​GTT​GAG​ATT​GTT
  iNOS
    Forward: GTT​CTC​AGC​CCA​ACA​ATA​CAA​GA
    Reverse: GTG​GAC​GGG​TCG​ATG​TCA​C
  GAPDH
    Forward: AGG​TCG​GTG​TGA​ACG​GAT​TTG
    Reverse: TGT​AGA​CCA​TGT​AGT​TGA​GGT​CA

Each experiment was repeated twice, and the gene expression differences between different groups were analyzed by the relative quantitative 2^−ΔΔCT^ method.

### Statistical Analysis

A majority of data obtained was analyzed by SPSS 22.0 and presented in Mean ± SD. Data variance was assessed by using one-way analysis of variance (ANOVA) (groups ≧3, Kolmogorov-Smirnov was performed before ANOVA analysis to justify the normal distribution character) or unpaired t test (groups = 2). For the analysis of survival rate, the Kaplan-Meier cumulative survival method and the differences among groups were analyzed by Log-rank (Mantel-Cox) test. Differences with *p* values less than 0.05 were considered statistically significant.

## Results

### The Expression of Gal-9 on Murine MSCs Was Enhanced by IFN-γ Stimulation

Adipose derived mesenchymal stem cells (AD-MSCs) exhibit a spindle fibroblast-like morphology (Supplementary Figure S1C). They did not express CD31 or CD45 but expressed CD73 and CD105 surface markers (Supplementary Figure S1D).

**FIGURE 1:**
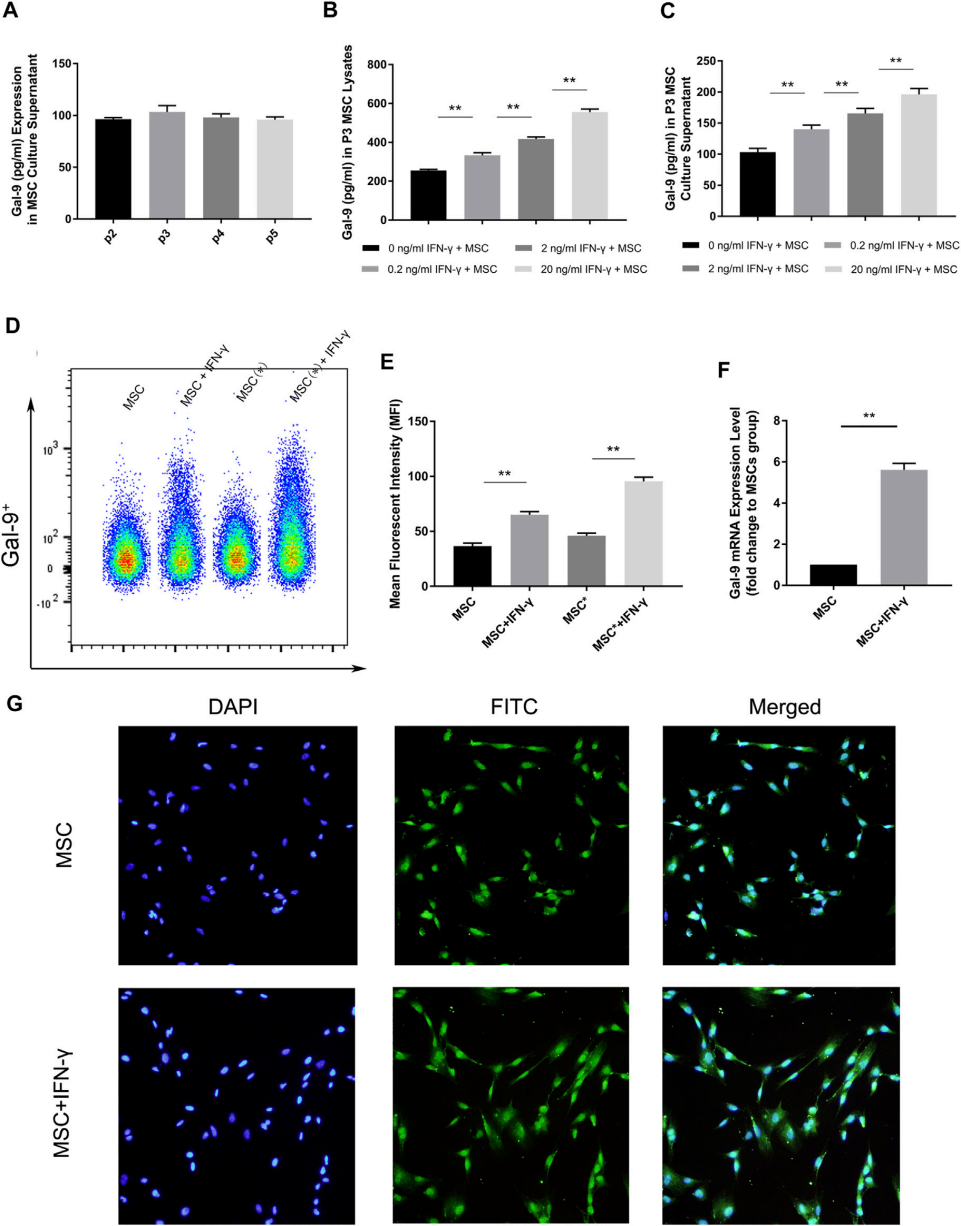
IFN-γ stimulation could enhance Gal-9 expression in AD-MSCs. **(A)** Gal-9 expression in second to fifth generation MSC supernatant. *n* = 3. **(B–C)** Gal-9 expression in third generation MSC lysate and culture supernatant increased in a dose-dependent manner by IFN-γ stimulation. *n* = 3. *p < 0.05, **p < 0.01. **(D–E)** Fluorescence intensity of Gal-9 in different samples (* indicates: permeabilization and fixation optimized before fluorescent antibody staining). **(F)** Gal-9 mRNA relative expression in IFN-γ pre-stimulated MSCs (20 ng/ml IFN-γ, 72 h) and non-stimulated MSCs. *n* = 3. *p < 0.05, **p < 0.01. **(G)** Representative immunofluorescence figures of MSCs with IFN-γ pre-stimulation (20 ng/ml, 72 h) or not. The nucleus was presented as blue and Gal-9 protein appeared as green. Bar graphs represent mean ± SD. Abbreviation: Gal-9, Galectin-9; p2-p5, passage 2-passage 5; MSC, mesenchymal stem cell.

Gal-9 expression in second to fifth generation AD-MSCs was measured by ELISA. By comparing with the standard sample, it was confirmed that Gal-9 was expressed in MSC culture supernatant (Figure 1A, *n* = 3). However, there was no statistical difference between different generations (P2 vs*.* P3, *p* = 0.057; P3 vs*.* P4, *p* = 0.17; P4 vs*.* P5, *p* = 0.43). Intriguingly, we found that Gal-9, expressed in MSCs (Figure 1B) and culture supernatant (Figure 1C), was significantly increased in a dose dependent manner with the stimulation of IFN-γ (*n* = 3, *p < 0.05, and **p < 0.01).

To further clarify the difference of Gal-9 expression, AD-MSCs with or without IFN-γ stimulation (20 ng/ml IFN-γ, 72 h) were prepared. Through flow cytometry analysis, it was found that the fluorescence of Gal-9 on MSCs increased apparently after IFN-γ stimulation (Figure 1D, *n* = 3). Moreover, the fluorescence intensity further improved by fixation and permeabilization process (Figure 1D, “*” indicates: permeabilization and fixation optimized before fluorescent antibody staining). Mean fluorescent intensity (MFI) evaluation confirmed the above findings (Figure 1E). Meanwhile, we performed RT-PCR analysis and found that Gal-9 mRNA expression in IFN-γ pre-stimulated MSCs was also significantly improved (Figure 1F, *n* = 3, *p < 0.05, and **p < 0.01). In addition, we also measured Gal-9 expression in IFN-γ pre-stimulated MSCs at 24 h, and found the upregulation of Gal-9 in AD-MSCs by ELISA and RT-PCR (Supplementary Figures S1A,B).

The quantitative-based detection method is still not intuitive. Therefore, we performed immunofluorescence staining (Figure 1G). As shown, we discovered that MSCs had a small amount of Gal-9 expression. But after IFN-γ stimulation, Gal-9 expression has a marked increase. Given these results, we speculated that Gal-9 was expressed both in the MSC surface and cytoplasm.

### Gal-9 Mediates MSC-Based Therapy in Ameliorating Endotoxemia Symptoms

**FIGURE 2:**
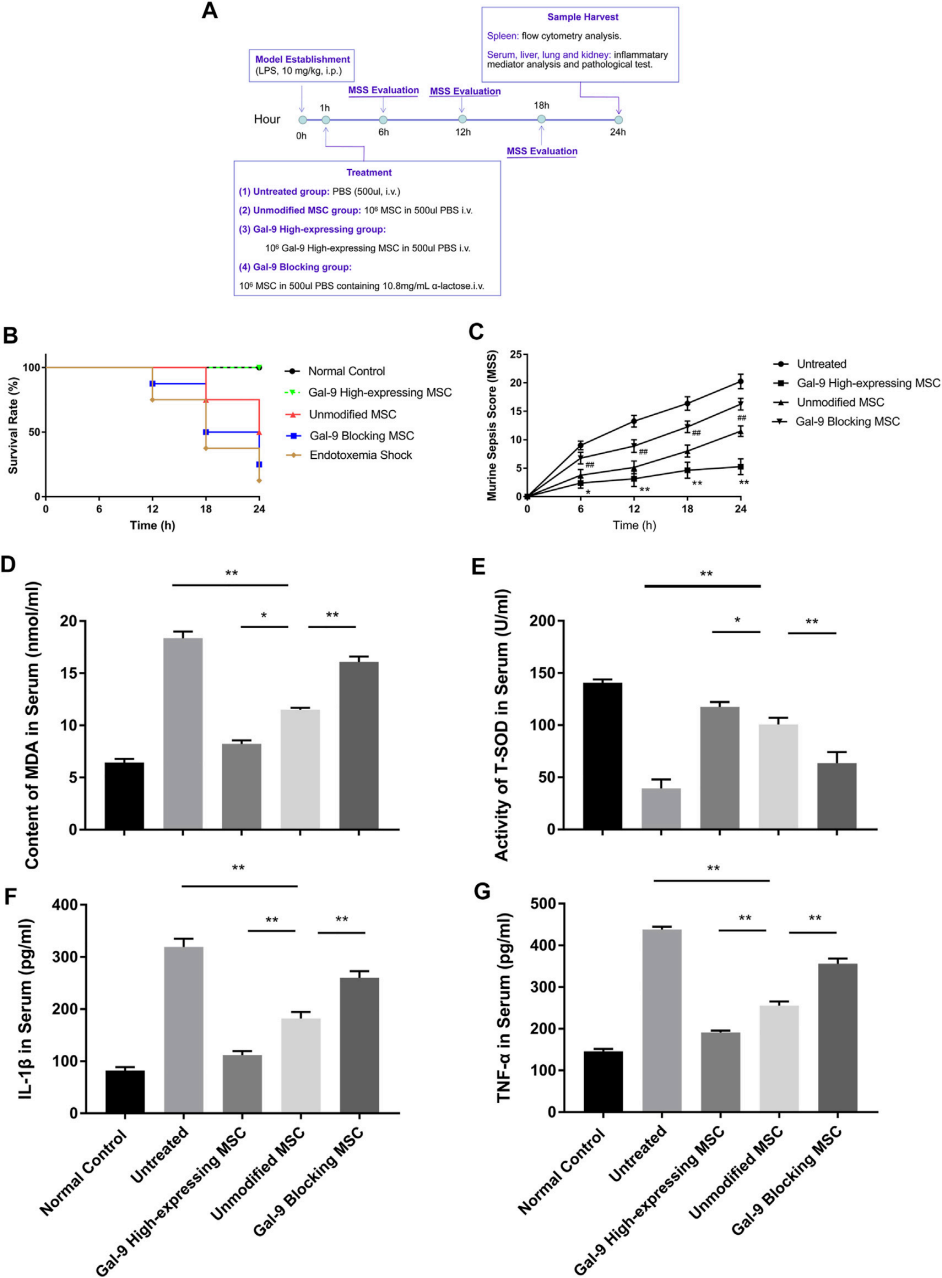
Gal-9 mediates MSC-based therapy in alleviating symptoms and inflammatory mediators’ expression. **(A)** Time schedule of this whole experiment. **(B)** Endotoxemia mice survival rate, *n* = 8. **(C)** Mean murine sepsis score (MSS) in different groups (*n* = 8, *p < 0.05, **p < 0.01, #*p* < 0.05, and ##*p* < 0.01). **(D–E)** MDA and T-SOD expression level in serum, *n* = 6. **(F–G)** IL-1β and TNF-α secretions in serum, *n* = 6. Data variance was assessed by using one-way analysis of variance (ANOVA), followed by least significant difference (LSD) test. *p < 0.05, **p < 0.01. Abbreviations: MDA, malondialdehyde; T-SOD, total superoxide dismutase.

To explore the involvement of Gal-9 in mediating the therapeutic effect of MSCs on endotoxemia, two investigators monitored the health conditions of endotoxemia mice (*n* = 8) and evaluated murine sepsis score (MSS) every 6 h. As shown in Figure 2B, the survival rate in the endotoxemia shock group was the lowest and remained lower in the Gal-9 blocking MSC group. Whereas it tends to be higher in the unmodified MSC group, but without statistical significance (vs*.* untreated group, *p* = 0.065). Interestingly, there was no death found in Gal-9 high-expressing MSC group at the end of observation (vs*.* unmodified MSC group, *p* = 0.025; vs*.* untreated group, *p* = 0.001).

When it turns to MSS, it was evaluated mainly based on the following aspects: appearance, spontaneous activity, eyes condition, level of consciousness, response to stimuli, respiration rate, and respiration quality. As shown, Gal-9 high-expressing MSC group had the lowest score, which was statistically different from the unmodified MSC group at different time points (Figure 2C, *n* = 8, #*p* < 0.5, and ##*p* < 0.01), while the Gal-9 blocking MSC group had a higher average score than the unmodified MSC group (*n* = 8, *p < 0.5, and **p < 0.01). Given the above findings, it indicates that Gal-9 is required for MSCs to alleviate endotoxemia symptoms. While after blocking Gal-9 functional receptor, the therapeutic effect of MSCs tends to be antagonized.

### Gal-9 Mediates MSC-Based Therapy in Alleviating Inflammatory Mediators Release in Endotoxemia

Based on the symptom divergence observed above, to further analyze oxidative stress and inflammatory factor secretion changes, we measured inflammatory mediators in serum. Malondialdehyde (MDA) is a metabolic product of lipid peroxidation, whose expression could represent the degree of cell damage. As shown in Figure 2D, it was found that MDA expression was lower in Gal-9 high-expressing MSC group, but significantly higher in the Gal-9 blocking MSC group (Figure 2D, Gal-9 high-expressing group vs. unmodified MSC group, *p* < 0.05; unmodified MSC group vs*.* Gal-9 blocking group, *p* < 0.01). Moreover, total superoxide dismutase (T-SOD) was recognized as the vital free radical scavenger. As shown in Figure 2E, we found that T-SOD increased significantly in the Gal-9 high-expressing MSC group, while decreased in Gal-9 blocking MSC group (Figure 2E, Gal-9 high-expressing MSC group vs. unmodified MSC group, *p* < 0.05; unmodified MSC group vs*.* Gal-9 blocking MSC group, *p* < 0.01).

Meanwhile, we have also analyzed IL-1β and TNF-α expressions in serum, both of which are pro-inflammatory factors and play a vital role in promoting sepsis. We found that there was a lower level of pro-inflammatory factors in the Gal-9 high-expressing MSC group (Figures 2F,G, vs*.* unmodified MSC group, IL-1β, *p* < 0.01; TNF-α, and *p* < 0.01). But after abolishing the Gal-9 suppressive effect, the mitigation effect was suppressed (Figures 2F,G, unmodified MSC group vs*.* Gal-9 blocking MSC group, IL-1β, *p* < 0.01; TNF-α, *p* < 0.01). In summary, it suggests that Gal-9 is actively involved in the modulating effects of MSCs in regulation of oxidative metabolites and inflammatory factors.

### Gal-9 Mediates MSC-Based Therapy in Regulation M1- and M2-type Macrophage Polarization

**FIGURE 3:**
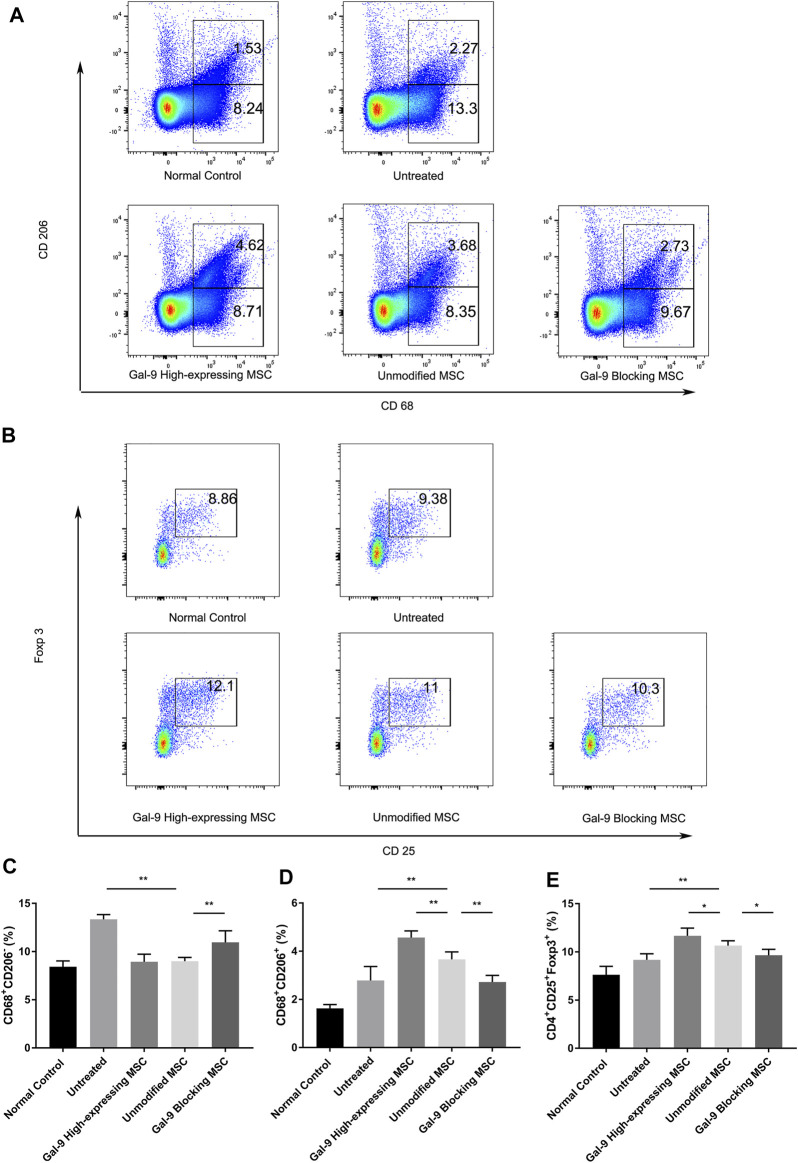
Gal-9 mediates MSC-based therapy in regulation of macrophage polarization and Tregs percentage. Spleen cells were collected 24 h after LPS injection, followed by staining with anti-mouse CD68 antibody for total macrophage, CD68^+^CD206^+^ for M2-type macrophage, and CD4^+^CD25^+^Foxp3^+^ for Tregs. **(A)** Representative dot plots of M1 (CD68^+^CD206^−^) and M2 (CD68^+^CD206^+^) macrophage. **(B)** Representative dot plots of Treg (CD4^+^CD25^+^Foxp3^+^). **(C)** Percentage of M1 (CD68^+^CD206^−^) macrophage. **(D)** Percentage of M2 (CD68^+^CD206^+^) macrophage. **(E)** Percentage of Tregs in spleen. Data variance was assessed by using one-way analysis of variance (ANOVA), followed by least significant difference (LSD) test, *n* = 6. *p < 0.05, **p < 0.01. Abbreviations: Treg, regulatory T cells; M total, total macrophage.

Macrophages are centrally involved in the pathogenesis of endotoxemia. However, whether Gal-9 high-expressing MSCs could affect the ratio of macrophage subtype is still unclear. Thus, we detected the percentages of M1- and M2-type macrophage (CD68^+^CD206^+^) ratio changes in splenocytes. Representative dot plots are shown in Figure 3A, and percentages of M1-type and M2-type macrophage are shown in Figures 3C,D. The proportion of M1 subtype cells was increased significantly in the untreated group, when compared with the control group (*p* < 0.01). In addition, the percentage of M1 cells in unmodified MSC and Gal-9 high-expressing group was decreased (vs. Gal-9 blocking group). However, there was no statistic difference between unmodified MSC and Gal-9 high-expressing group (*p* = 0.21).

Furthermore, the proportion of M2-type macrophage in the unmodified MSC treated group was significantly higher than that in the untreated group (*p* < 0.01, *n* = 6). In addition, when the expression of Gal-9 was increased or antagonized, respectively, the proportion of M2 cells was shown higher or lower than that in the unmodified MSC group (Figure 3D, Gal-9 high-expressing MSC group vs*.* unmodified MSC group, *p* < 0.01; unmodified MSC group vs*.* Gal-9 blocking MSC group, *p* < 0.01). Given the above results, it suggests that the Gal-9 high-expressing MSCs participate in the regulation of macrophages and promote macrophage polarization toward M2-subtype.

### Gal-9 Mediates MSC-Based Therapy in Boosting Tregs Generation

Tregs have a role in inhibiting T cell proliferation and cytokine production, thereby mediating the maintenance of immunologic homeostasis. Treg proportion in splenocytes (CD4^+^CD25^+^Foxp3^+^) was also detected in our experiment. Representative dot plots were displayed in Figure 3B and percentages of Tregs were shown in Figure 3E. The proportion of Tregs in the untreated group was a little bit higher than that in the normal control group, which is in accordance with previous reports (Scumpia et al., 2006). In the unmodified MSC treated group, the proportion of Tregs was higher than that of the untreated group (*p* < 0.01, *n* = 6). While after elevating Gal-9 expression in MSCs, the Treg population was significantly increased (Gal-9 high-expressing MSC group vs*.* unmodified MSC group, *p* < 0.05). In summary, it indicates that Gal-9 is involved in MSC immunomodulatory effect by regulating Treg proportions.

### Gal-9 Mediates MSC-Based Therapy in Attenuating Hepatic Damage

**FIGURE 4:**
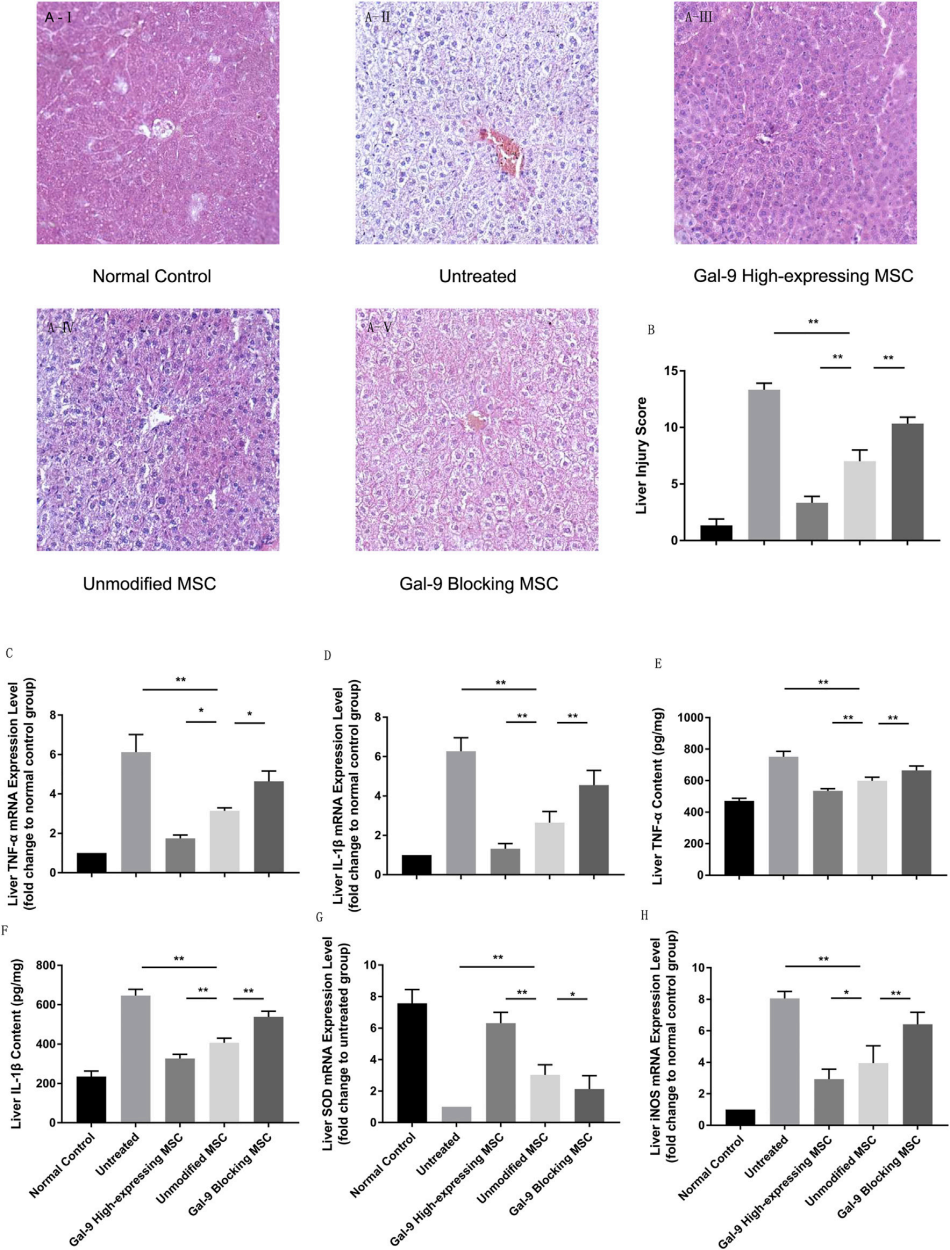
Gal-9 mediates MSC-based therapy in attenuating hepatic damage. **(A)** Photomicrographs of representative histological sections (H&E staining, original 100 × magnification) of liver in each group. **(B)** Liver injury score. Double-blind reviewing was evaluated by the following criteria: necrosis, sinus congestion and edema, lipid vacuoles and infiltration of hyperemia, and inflammatory cells (0–4, total: 0–16). **(C)** Liver TNF-α mRNA expression level. **(D)** Liver IL-1β mRNA expression level. **(E)** TNF-α protein concentration in liver homogenate. **(F)** IL-1β protein concentration in liver homogenate. **(G)** Liver SOD mRNA expression level. **(H)** Liver iNOS mRNA expression level. Data variance was assessed by using one-way analysis of variance (ANOVA), followed by least significant difference (LSD) test, *n* = 6. *p < 0.05, and **p < 0.01. Abbreviations: iNOS, inducible nitric oxide synthase; SOD, superoxide dismutase.

The liver is one of the most vulnerable organs in the systemic inflammatory response syndrome (SIRS). We analyzed liver pathological manifestations, and a representative picture (100×) is displayed in Figure 4A. As shown, in the untreated group, hepatic damage was the most severe, which was displayed with central venous sinus congestion, lipoid vacuoles, hyperemia cells infiltration, and lobular inflammation. However, the pathological manifestation seemed with relief in the unmodified MSC treated group, which was manifested as the disappearance of central venous sinus congestion and the reduction of lipid vacuoles, but there were still hyperemia cells and inflammatory cells infiltration among the hepatic lobules. This pathological manifestation was markedly relieved in Gal-9 the high-expressing MSC treated group. But after blocking Gal-9 suppressive effect, sinus congestion, and hepatocyte vacuolation, injury was displayed again.

We further measured cytokine mediator changes in liver homogenate. TNF-α and IL-1β were considered as essential factors for the function of the M1-type macrophage and detected in serum previously. To further explore these cytokine changes in functional organs, we measured their relative mRNA expression levels. As shown in Figures 4C,D, TNF-α and IL-1β mRNA expression increased significantly in the untreated group, but decreased in the unmodified MSC treatment group (untreated group vs*.* unmodified MSC group, *p* < 0.01). Additionally, after giving the Gal-9 high-expressing MSCs, TNF-α and IL-1β further decreased (vs*.* unmodified MSC group, TNF-α, *p* < 0.05; IL-1B, *p* < 0.01). Furthermore, liver protein homogenates were collected, and we also measured TNF-α and IL-1β by ELISA. Consistently, we found that the protein expression changes of TNF-α and IL-1β were consistent with the mRNA alternation (Figures 4E,F).

To explore oxidative stress levels, we also analyzed iNOS and SOD expression changes in liver. We found that SOD mRNA change was in line with the trends indicated in serum, which was significantly higher in the Gal-9 high-expressing MSC group (Figure 4G, vs*.* the unmodified MSC group, *p* < 0.01). However, iNOS, an indicator for NO synthesis which promotes injury, decreased in the unmodified MSC treated group (Figure 4H, vs*.* untreated group, *p* < 0.01), and further reduced in the Gal-9 high-expressing MSC group (vs*.* unmodified MSC group, *p* < 0.05). Combined with the above results, it suggests that the Gal-9-mediated MSC therapy could alleviate liver pathological damage and reduce inflammatory factor expressions in liver tissues, which is consistent with the trend in serum.

### Gal-9 Mediates MSC-Based Therapy in Alleviating Pulmonary Injury

**FIGURE 5:**
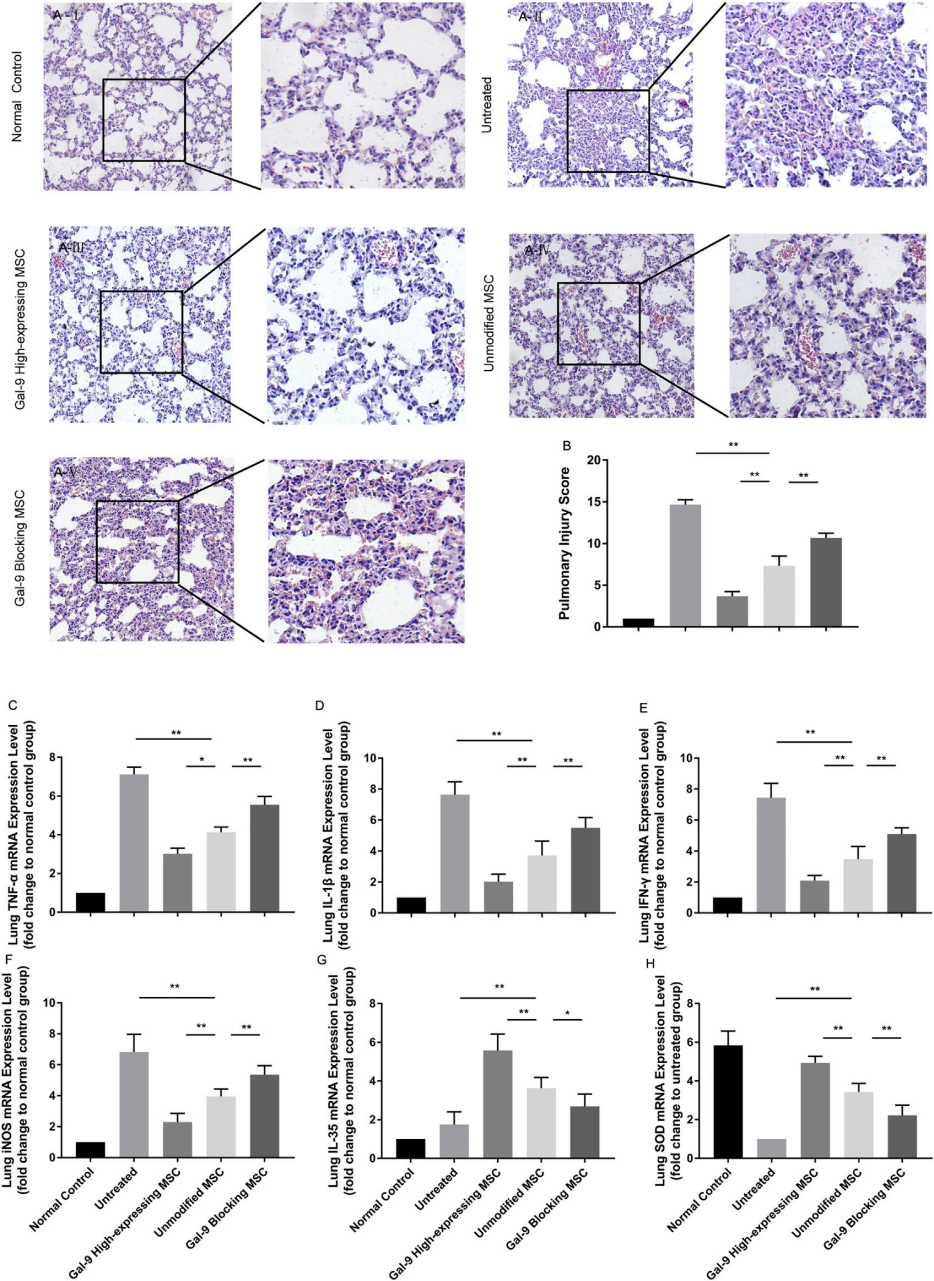
Gal-9 mediates MSC-based therapy in alleviating pulmonary injury. **(A)** Representative histological sections of mouse lung in each group (H&E, 100 × magnification) and a selected small square area were magnificently displayed on the right side (200 × magnification). **(B)** Pulmonary injury score. Criteria for evaluating lung injury were as follows: edema, alveolar and interstitial inflammation, alveolar and interstitial hemorrhage, and necrosis (0−4, total: 0–16). **(C–H)** mRNA expression levels of TNF-α **(C)**; IL-1β **(D)**; IFN-γ **(E)**; iNOS **(F)**; EBI3 (IL-35) **(G)**; SOD **(H)**. Data variance was assessed by using one-way analysis of variance (ANOVA), followed by least significant difference (LSD) test, *n* = 6. *p < 0.05, **p < 0.01. Abbreviations: EBI3, Epstein-Barr virus-induced gene3; iNOS, inducible nitric oxide synthase; SOD, superoxide dismutase.

We further analyzed the inflammatory mediator expressions and pathological changes in lung. As displayed in Figure 5A, in the untreated group, it appeared with obvious alveolar and interstitial inflammation, hemorrhage, and necrosis. However, after treatment with unmodified MSCs, the inflammatory infiltration and tissue necrosis tend to relieve. The injury further exacerbated in the Gal-9 blocking MSC group, and significantly alleviated in Gal-9 high-expressing MSC group. The pathological score based on pulmonary pathological manifestation is shown in Figure 5B.

In addition, TNF-α, IL-1β, IFN-γ, and iNOS mRNA expressions in the lung tissue were evaluated. As expected, TNF-α, IL-1β, IFN-γ, and iNOS were both decreased in the Gal-9 high-expressing MSC treated group (Figures 5C–F, vs*.* unmodified MSC group, TNF-α, *p* < 0.05; IL-1β, IFN-γ, and iNOS, *p* < 0.01), but increased in the Gal-9 blocking MSC group (vs*.* unmodified MSC group, *p* < 0.01). Meanwhile, we also measured IL-35 and SOD. It turned out that IL-35 and SOD mRNA expressions were little higher in the Gal-9 high-expressing MSC group (Figures 5G,H, vs*.* unmodified MSC group *p* < 0.01), and lower in the Gal-9 blocking MSC group (vs*.* unmodified MSC group, IL-35, *p* < 0.05; SOD, and *p* < 0.01). In total, the above results indicate that Gal-9 mediates MSC-based therapy in relieving lung pathological damage and reducing damaging factor expressions.

### Gal-9 Plays a Role in MSC-Mediated Therapy in Lightening Renal Damage

**FIGURE 6:**
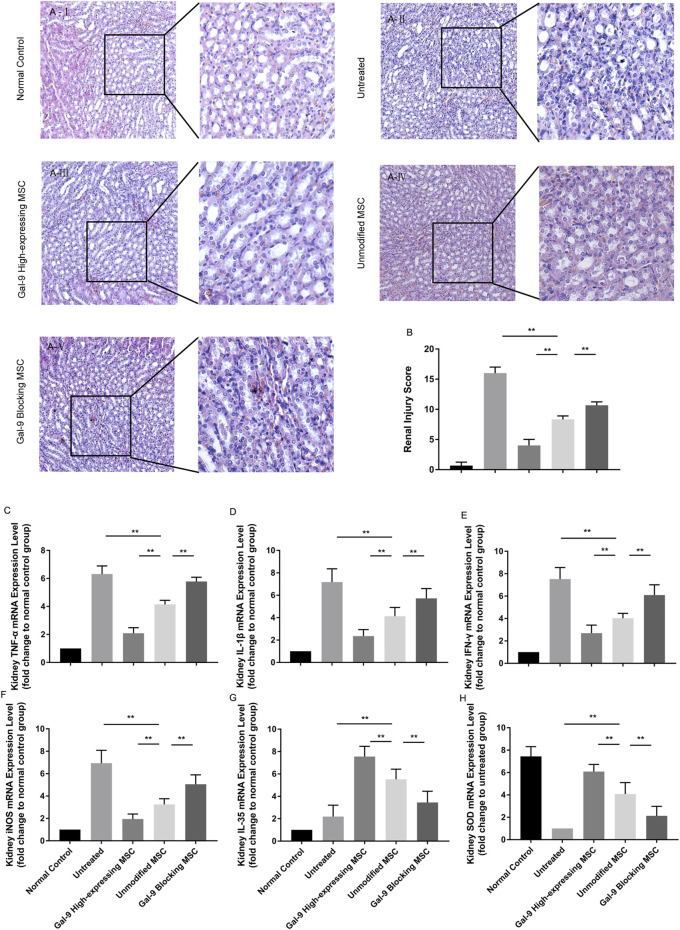
Gal-9 Plays a role in MSC-mediated therapy in lightening renal damage. **(A)** Representative histological sections of mouse kidney in each group (H&E, 100 × magnification). To specifically observe the difference, a selected small square area was magnificently displayed on the right side (200 × magnification). **(B)** Renal injury score. Criteria for assessing kidney injury are as follows: tubular dilatation/flattening, tubular casts, and tubular degeneration/vacuolization (cortex and medulla, 0–3, total: 0–18). **(C–H)** mRNA expression levels of TNF-α **(C)**; IL-1β **(D)**; IFN-γ **(E)**; iNOS **(F)**; EBI3 (IL-35) **(G)**; SOD **(H)**. Data variance was assessed by using one-way analysis of variance (ANOVA), followed by least significant difference (LSD) test, *n* = 6. *p < 0.05, **p < 0.01. Abbreviations: EBI3, Epstein-Barr virus-induced gene3; iNOS, inducible nitric oxide synthase; SOD, superoxide dismutase.

Furthermore, we obtained the kidneys and evaluated damages among different groups. As shown in Figure 6A, the kidneys in the untreated group showed obvious tubular casts, inflammatory cell infiltration, tubular degeneration, and vacuolization in the cortex and medulla. While the injury tends to be relieved in the unmodified MSC treated group and normalized in the Gal-9 high-expressing MSC group, which is only with a small amount of tubular dilatation. The corresponding pathological scores were shown in Figure 6B.

Total RNA of kidney tissue was extracted to assess inflammatory factor changes. As shown, TNF-α, IL-1β, IFN-γ, and iNOS mRNA expressions were significantly lower (Figures 6C–F, *p* < 0.01), while IL-35 and SOD were significantly higher (Figures 6G,H, *p* < 0.01) in the unmodified MSC group (vs*.* untreated group). Moreover, TNF-α, IL-1β, IFN-γ, and iNOS mRNA expressions were further reduced (*p* < 0.01), while IL-35 and SOD expressions were increased (vs*.* unmodified MSC group, *p* < 0.01) in the Gal-9 high-expressing MSC group. Notably, the above immunomodulatory effect was weakened when Gal-9 was antagonized by α-lactose (Gal-9 blocking MSC vs*.* unmodified MSC group, *p* < 0.01). Given the above results, it suggests that Gal-9 plays an important role in MSC-based therapy, and especially in alleviating renal damages.

### Gal-9 Highly Expresses in Liver, Lung, and Kidney After Gal-9 High-Expressing MSC Treatment

**FIGURE 7:**
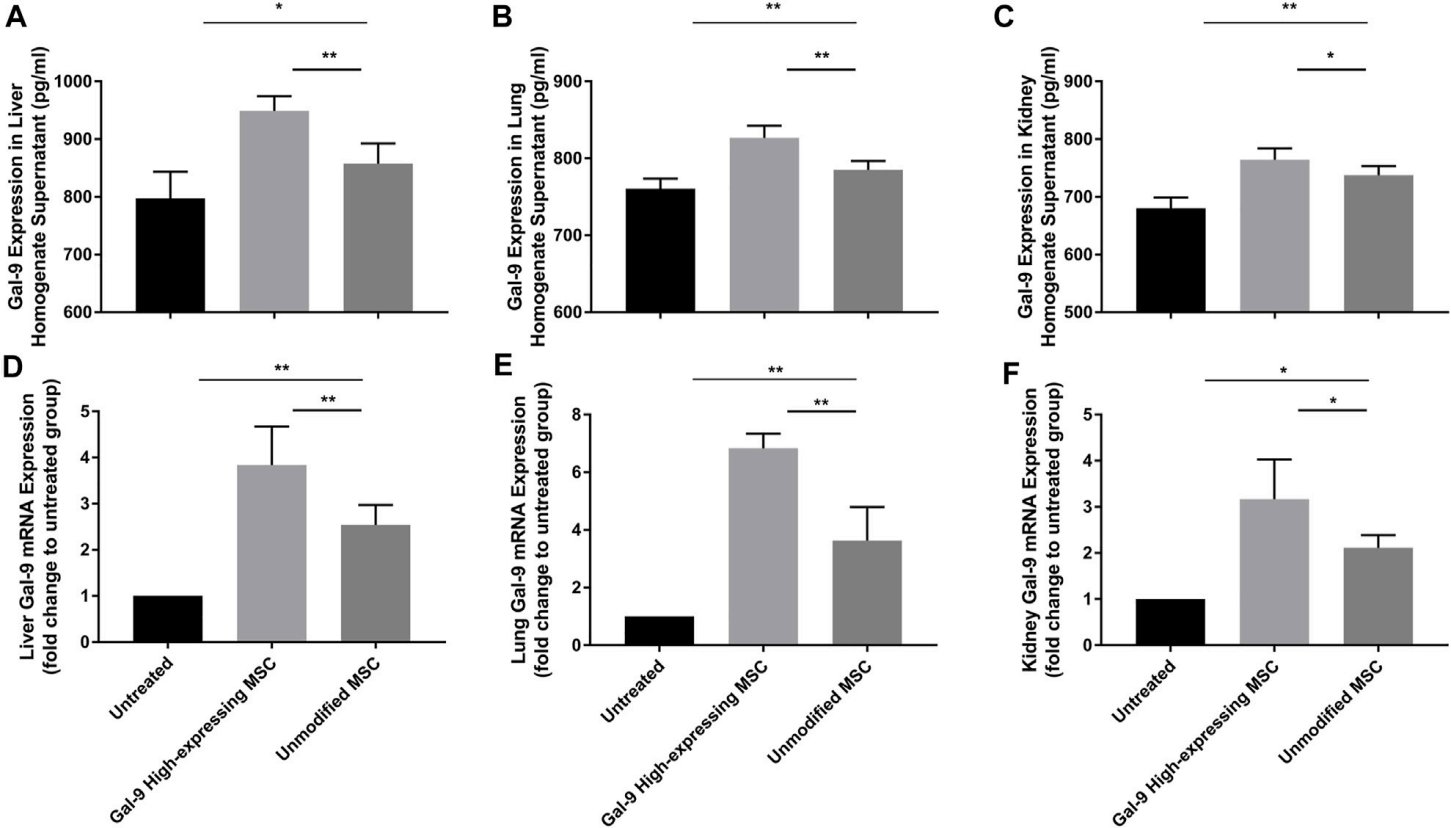
Gal-9 highly expresses in liver, lung, and kidney after Gal-9 high-expressing MSC treatment. **(A–C)** Gal-9 protein expression in the homogenate of liver **(A)**, lung **(B),** and kidney **(C)**, *n* = 6. **(D–F)** Relative Gal-9 mRNA expression levels in liver **(D)**, lung **(E)**, and kidney **(F)**, and *n* = 6. Data variance was assessed by using one-way analysis of variance (ANOVA), followed by least significant difference (LSD) test. *p < 0.05, **p < 0.01.

We have also measured Gal-9 expression in liver, kidney, and lung homogenates (Figures 7A–F). As shown, after administration with unmodified MSCs, Gal-9 expression was increased at both the protein and the mRNA levels (vs. untreated group; protein: liver, *p* < 0.05; lung, *p* < 0.01; kidney, *p* < 0.01; mRNA: liver, *p* < 0.01; lung, *p* < 0.01; kidney, *p* < 0.05). Moreover, Gal-9 expression was further improved after treatment with Gal-9 high-expressing MSCs (vs. unmodifified MSC group, protein: liver, *p* < 0.01; lung, *p* < 0.01; kidney, *p* < 0.05; mRNA expression: liver, *p* < 0.01; lung, *p* < 0.01; kidney, *p* < 0.05). Based on the above results, it suggests that Gal-9 high-expressing MSC treatment assists in enhancing Gal-9 expression in the target organs, and the higher level of Gal-9 was supposed to participate in the repair of organ damages.

Based on the results from previous studies, it has already been confirmed that α-lactose was an antagonist of Gal-9, which could successfully block Gal-9 binding (Ungerer et al., 2014). Therefore, Gal-9 expression in the Gal-9 blocking MSC group was not shown in Figure 7.

## Discussion

Endotoxemia is mediated by irregular and aberrant inflammatory response toward LPS, which could result in multiple organ dysfunction syndrome (MODS), and death (Llewelyn and Cohen, 2002), while the current treatment strategies in clinic are still limited. MSCs have been used in the treatment of multiple immune dysregulated diseases and exhibited promising advantages (Weil et al., 2010). However, mechanisms that govern MSC capability in modulating immune response are incomprehensive.

Growing evidence has suggested that MSCs could modulate the immune response through cell-to-cell contact or cytokine secretion, such as IL-10, TGF-β, VEGF, PGE2, and indoleamine-2,3-dioxygenase (IDO) (Kang et al., 2008; Spees et al., 2016). There were also reports demonstrating that IFN-γ could improve MSC immuno-regulation efficacy, but the mechanism underlined is rarely explored (Krampera et al., 2006; Polchert et al., 2008). It has been previously reported by Ungerer et al. that IFN-γ stimulation significantly increased Gal-9 mRNA expression in MSCs, but had a minor effect on affecting other markers, including CXCR1, CXCR2, CXCR4, CXCR5, CXCR7, Gal-1, Gal-2, Gal-3, Gal-4, Gal-7, Gal-8, Gal-12, and Gal-13 (Ungerer et al., 2014). In line with their findings, we also confirmed that IFN-γ pre-stimulation could promote Gal-9 expression in MSCs, and Gal-9 was actively involved in the therapeutic effects of MSCs in alleviating endotoxemia.

Gal-9, a member of the β-galactoside-binding galectin family, was recognized as the T cell immunoglobulin-3(Tim-3) ligand (Cao et al., 2007). TIM-3, expressed on Th1, Th17, CD8 T, macrophage, and natural killer (NK) cells, has been identified as a regulatory molecule (Anderson et al., 2016). Recent studies have shown that Gal-9 signaling could negatively regulate Th1 and Th17 immunity through Gal-9/Tim-3 interaction (Zhu et al., 2005; Seki et al., 2008). Moreover, there were other reports demonstrating that Gal-9 could promote macrophage polarization to M2 subtype (Lv et al., 2017; Li et al., 2018) and boost Foxp3^+^ Treg generation (Seki et al., 2008). However, the *in vivo* studies concerning Gal-9-mediated immunoregulation of MSCs are still scanty.

In the current study, through the experimental verification at the protein and gene level, we found that Gal-9 expression in MSCs increased significantly with IFN-γ prestimulation. Then, the endotoxemia model was induced by LPS intraperitoneal (i.p.) injection (10 mg/kg) according to the previous reports (Zhai and Guo, 2016). Gal-9 high-expressing MSCs, unmodified MSCs, and Gal-9 blocking MSCs were given to mice, respectively. It is worthy to notice that α-lactose, reported as the specific antagonist of Gal-9, was applied to block Gal-9 binding effect in large numbers of previous studies (Ungerer et al., 2014). In this study, we also chose α-lactose as an antagonist for blocking the effect of Gal-9 but did not evaluate the abrogating effect of α-lactose on other galectins besides galectin-9.

In this study, we found that Gal-9 high-expressing MSCs did have certain positive significance in improving the survival rate of endotoxemia mice. Murine sepsis score (MSS) further confirmed the above findings, which is the lowest in the Gal-9-mediated MSC treatment group, indicating the best therapeutic effect involved in this group.

TNF-α and IL-1β, majorly secreted by macrophages, were recognized as essential initiators in endotoxemia shock (Tang et al., 2010). When administration of anti-TNF-α Abs or knocking-out of TNFR, it could greatly diminish or abrogate mortality in endotoxic models (Ungerer et al., 2014; Wang et al., 2003; Saitoh et al., 2008). In our experiment, circulating TNF-α and IL-1β were also detected. Through comparison, we have found that Gal-9 high-expressing MSC treatment group has an overall lower inflammation level than that of the Gal-9 antagonistic group.

In addition, circulating oxidative stress factors were also measured in the present study. Oxidative stress was reported to participate in the pathogenesis of endotoxemia and contribute to multiple organ failure in septic patients (Boueiz and Hassoun, 2009; Bedreag et al., 2015). Anti-oxidant management focusing on mimic superoxide dismutase enzyme activity has been shown with univocal effect in preventing cellular energetic failure (Salvemini and Cuzzocrea, 2002). Thus, we speculate that whether improving Gal-9 expression in MSCs could also have a modulating role in relieving oxidative stress damage. Based on this, we have measured SOD and MDA expressions in endotoxemia mice serum. As expected, we found that Gal-9 high-expressing MSCs had an obvious effect in downregulating circulating MDA level, but upregulating SOD expression.

Macrophages were generally believed to be categorized into two phenotypic subsets: M1 pro-inflammatory subtype and M2 selectively activated anti-inflammatory subtype (Mills et al., 2000). Macrophages are highly versatile phagocytic cells whose diverse effector functions can be selectively reprogrammed by an array of environmental signals (Hume, 2015). IFN-γ, TNF-α, and LPS could promote macrophages to differentiate into the M1 subtype. Whereas exposure to IL-4 or IL-10 could promote macrophages to differentiate into the M2 subtype (Wang et al., 2014; Shapouri-Moghaddam et al., 2018). There are reports suggesting that treating septic mice with MSCs could promote macrophage polarization to IL-10 production type, rather than proinflammatory subtype (Németh et al., 2009). Additional investigations revealed that increasing Gal-9 expression in Raw 264.7 cell could promote macrophage polarization to the M2 phenotype (Lv et al., 2017). However, whether Gal-9 high-expressing MSCs could also affect macrophage polarization in endotoxemia is still unclear. In this study, we analyzed macrophage proportion and subtype in each group. We found that Gal-9-mediated MSC therapy could promote macrophage polarization into the M2 subtype. Meanwhile, through detecting TNF-α and IL-1β levels in serum and organ homogenates, it also indirectly reflects that the pro-inflammatory function of M1 type macrophages was inhibited. On the contrary, the anti-inflammatory effect of M2 type tended to be manifested.

Increased Tregs ratios have also been observed in septic patient serum, but their presence does not contribute significantly to overall survival (Scumpia et al., 2006; Delano and Ward, 2016). While controversially, some reports demonstrated that adoptive transfer of CD4^+^CD25^+^ Treg could promote bacterial clearance (Scumpia et al., 2007) and improve survival rate in polymicrobial sepsis mice (Heuer et al., 2005). Further experiments concerning deleting functional Tregs (CD25 KO mice) or implementing anti-CD25 monoclonal antibody could lead to acute death in an original nonlethal LPS administration (Okeke et al., 2013). While from our perspective, we hold that Treg is beneficial and well needed before immune storm formation. At the initial stage of endotoxemia, Treg is a prerequisite, similar with M2 subtype macrophages, to inhibit the development of inflammation. However, the immune response tends to be exhausted at the end stage of endotoxemia. Then, the increase of Treg and M2 transform into the suppressors and are thus harmful to the immune response.

Based on the above, we recommend Gal-9 high-expressing MSCs should be injected at early stages once endotoxemia tends to occur. In our experiment, we administrated Gal-9 high-expressing MSCs 1 h later after modeling and discovered that Treg population in the unmodified MSC-treated group was higher than that of the untreated group, and the effect of MSCs was strengthened by elevating Gal-9 expression and inhibited by Gal-9 antagonist.

Besides, we also measured the pathological changes of liver, kidney, and lung, and the mRNA expressions of inflammatory factors in tissue homogenates. According to the standards criteria (Smith et al., 1997; Wang et al., 2009; Inata et al., 2018), two pathologists evaluated the pathological manifestations of the specific organs. The statistical results revealed that there were severe damages in the untreated group. While in the unmodified MSC-treated group, the pathological damages tend to be alleviated. When it comes to the Gal-9 high-expressing MSC group, the injury was further relieved. But the therapeutic effect of MSCs was significantly abrogated by α-lactose antagonist. In addition, we evaluated the pro-inflammatory factors and oxidative stress mediators (TNF-α, IL-1β, IFN-γ, IL-35, iNOS, and SOD) in liver, lung, and kidney tissue homogenates. The results were consistent with the pathological findings, which further explained that Gal-9 was involved in the therapeutic effect of MSCs in alleviating organ damage.

Through cell tracking experiments, our group has previously verified that MSCs could survive in mice and migrate to damaged organs, such as liver, lung, and spleen (Lu et al., 2016). Based on the hypothesis that Gal-9 is critical for MSCs to alleviate organ damage, and to further investigate the expression differences of Gal-9 among organs, we have evaluated the protein and mRNA levels of Gal-9 in liver, lung, and kidney tissue homogenates. As expected, after administering unmodified MSCs, Gal-9 expression was discovered in the target organs. Moreover, this expression was further increased after infusion with Gal-9 high-expressing MSCs. Given above, we have reasons to believe that Gal-9 high-expressing MSC treatment assists in enhancing Gal-9 expression in the target organs and the higher level of Gal-9 participates in the repair of organ damage. In addition, it has been reported that treatment with Gal-9 could mediate the depletion of CD4^+^Tim-3^+^ cells (He et al., 2009; Sakai et al., 2011). Therefore, we speculate that treatment with Gal-9 high expressing MSCs would mediate the depletion of CD4^+^Tim-3^+^ cells.

Although these experiment results were inspiring and promising, the modeling method might not be ideal, in which injecting LPS could only simulate the pathophysiological changes of the inflammatory response imbalance in clinical sepsis (Dickson and Lehmann, 2019). To better mimic the occurrence and development of clinical sepsis, the participation of bacteria is required, and the cecum ligation and puncture (CLP) model seems to have more advantages (Rittirsch et al., 2009). In addition, the present study could only provide evidence, to an extent, in illustrating the role of Gal-9 in mediating the immunomodulatory effect of MSCs. The specific molecular mechanism of generating Gal-9 in pre-stimulated MSCs and Gal-9-Tim-3 downstream pathways remains unclear and needs further investigation. Knockout or virus transfection of the Gal-9 gene in MSCs will be performed in our next-step project, which would provide much solid evidence. The whole gene-wide sequencing of IFN-γ-stimulated MSCs is also required to evaluate other regulatory factors besides Gal-9. The present study indicates that Gal-9 is a vital factor for MSCs in alleviating endotoxemia injury, which will enlighten the following in-depth research on further evaluation of Gal-9 in mediating the effect of MSCs.

Furthermore, given the reports that immune exhaustion occurs in the late stage of endotoxemia (Delano and Ward, 2016), we recommend that MSCs should be given at an early time to limit and inhibit the initial inflammation response. At the same time, the experimental hypothesis, in which comparing the effects of MSC administration at different time points of sepsis, has also been proposed.

Taken together, this experiment has verified the significance of Gal-9-mediated MSC therapy in relief of endotoxemia, which mainly manifested as attenuating circulating pro-inflammatory mediator secretion, promoting macrophage polarization to M2-subtype, inducing the increase of Treg population, and facilitating the alleviation of multiple organ injury. Even though MSCs regulating the immune response through cell-to-cell contact or regulatory factors secretion (IL-10, TGF-β, VEGF, PGE2, and IDO) have been continuously verified, focusing on Gal-9-mediated MSC therapy is just beginning and staying at the *in vitro* experimental stage. Our work provides a novel idea for supplementing the research of MSC immunoregulatory mechanism. At the same time, it also forms a basis for the next step in-depth mechanism study and the potential clinical use in the treatment of endotoxemia.

## Data Availability

The original contributions presented in the study are included in the article/Supplementary Material, further inquiries can be directed to the corresponding author.

## Supplementary Material

Supplementary Figure S1
**(A–B)** Gal-9 expression in IFN-γ pre-stimulated MSCs (20 ng/ml) at 24-h. (**C–D**) Adipose derived mesenchymal stem cell (AD-MSC) morphology and marker expression. Data variance was assessed by using one-way analysis of variance (ANOVA), followed by least significant difference (LSD) test. **p* < 0.05, ***p* < 0.01.
