# Supplementary figures and images for: Galectin-9 Mediates the Therapeutic Effect of Mesenchymal Stem Cells on Experimental Endotoxemia
Source: Front Cell Dev Biol. 2022 Feb 17;10:700702. doi: 10.3389/fcell.2022.700702 (PMC8893172; doi:10.3389/fcell.2022.700702)

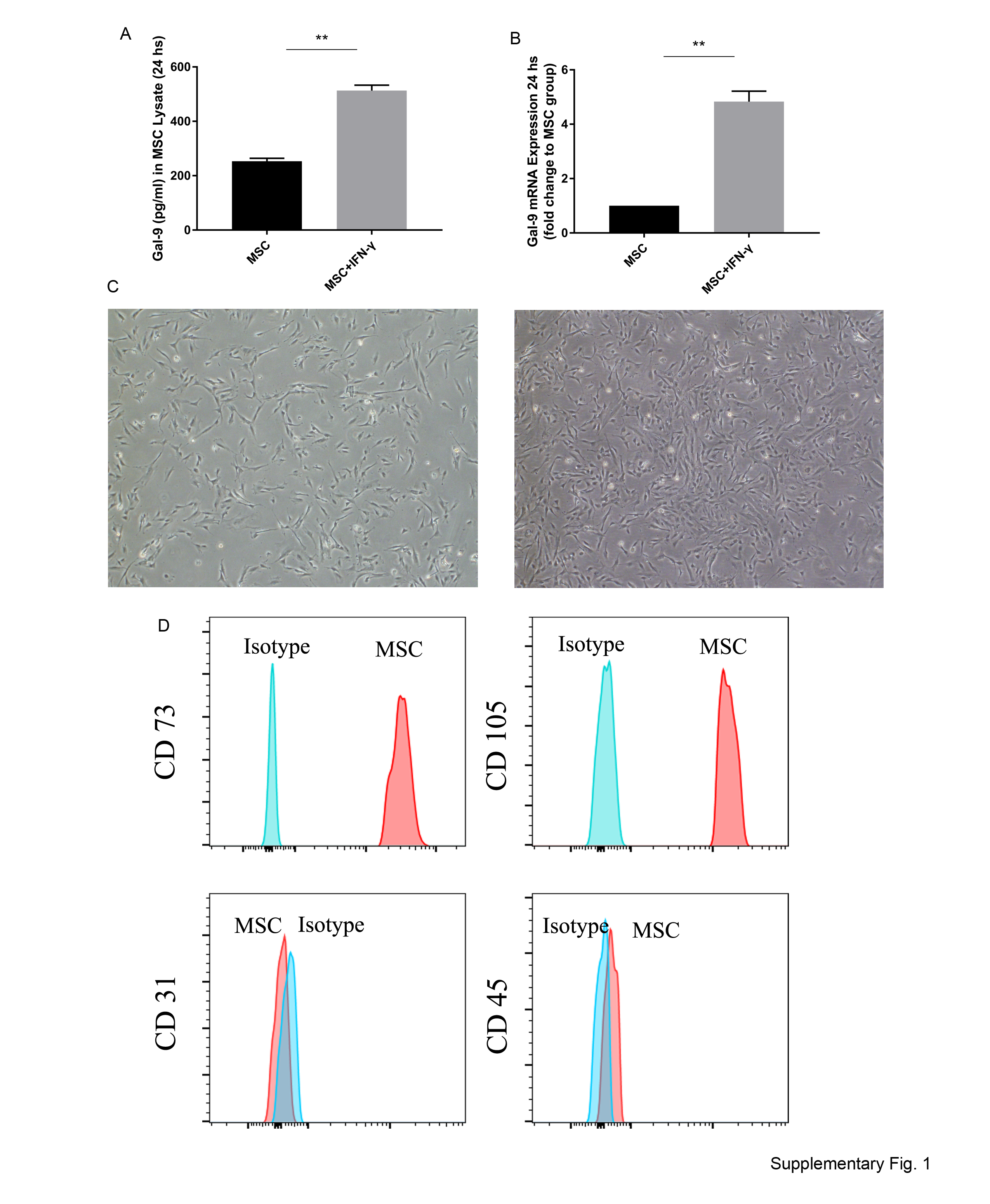

Supplement: Supplementary file 1 [file Image1.tif]
